# Asymmetry Analysis of Macular Inner Retinal Layers for Glaucoma Diagnosis: Swept-Source Optical Coherence Tomography Study

**DOI:** 10.1371/journal.pone.0164866

**Published:** 2016-10-20

**Authors:** Sang-Yoon Lee, Eun Kyoung Lee, Ki Ho Park, Dong Myung Kim, Jin Wook Jeoung

**Affiliations:** 1 Department of Ophthalmology, Seoul National University College of Medicine, Seoul, South Korea; 2 Department of Ophthalmology, Jeju National University Hospital, Jeju-si, South Korea; 3 Department of Ophthalmology, Seoul National University Hospital, Seoul, South Korea; Charite Universitatsmedizin Berlin, GERMANY

## Abstract

**Purpose:**

To report an asymmetry analysis of macular inner retinal layers using swept-source optical coherence tomography (OCT) and to evaluate the utility for glaucoma diagnosis.

**Design:**

Observational, cross-sectional study.

**Participants:**

Seventy normal healthy subjects and 62 glaucoma patients.

**Methods:**

Three-dimensional scans were acquired from 70 normal subjects and 62 open angle glaucoma patients by swept-source OCT. The thickness of the retinal nerve fiber layer, ganglion cell-inner plexiform layer (GCIPL), ganglion cell complex, and total retina were calculated within a 6.2×6.2 mm macular area divided into a 31×31 grid of 200×200 μm superpixels. For each of the corresponding superpixels, the thickness differences between the subject eyes and contra-lateral eyes and between the upper and lower macula halves of the subject eyes were determined. The negative differences were displayed on a gray-scale asymmetry map. Black superpixels were defined as thickness decreases over the cut-off values.

**Results:**

The negative inter-ocular and inter-hemisphere differences in GCIPL thickness (mean ± standard deviation) were -2.78 ± 0.97 μm and -3.43 ± 0.71 μm in the normal group and -4.26 ± 2.23 μm and -4.88 ± 1.46 μm in the glaucoma group. The overall extent of the four layers’ thickness decrease was larger in the glaucoma group than in the normal group (all *P*s<0.05). The numbers of black superpixels on all of the asymmetry maps were larger in the glaucoma group than in the normal group (all *P*s<0.05). The area under receiver operating characteristic curves of average negative thickness differences in macular inner layers for glaucoma diagnosis ranged from 0.748 to 0.894.

**Conclusions:**

The asymmetry analysis of macular inner retinal layers showed significant differences between the normal and glaucoma groups. The diagnostic performance of the asymmetry analysis was comparable to that of previous methods. These findings suggest that the asymmetry analysis can be a potential ancillary diagnostic tool.

## Introduction

Healthy organ pairs mostly show symmetric anatomic features. In healthy subjects, the retinal layers show significant symmetry between the right and left eyes. On the other hand, the occurrence and progression of clinical disease are often asymmetric between the right and left eyes. This opens up the possibility that evaluation of the inter-ocular symmetry of retinal layers can help detect glaucomatous changes. Several studies have reported the inter-ocular asymmetry of the peripapillary retinal nerve fiber layer (RNFL) thickness measured by optical coherence tomography (OCT). [1–6] However, the amount of difference was relatively small, and the difference showed inconsistency between studies, depending on the topographic area. A recent study showed significant inter-ocular symmetry of ganglion cell-inner plexiform layer (GCIPL) thickness measured by spectral-domain OCT in normal healthy eyes. [7] Sullivan-Mee et al. have evaluated diagnostic capabilities of inter-ocular differences in total macular thickness, and inter-ocular macular thickness asymmetry showed the highest diagnostic sensitivity between early glaucoma patients and controls. [8]

Furthermore, the distribution of retinal ganglion cell axons and cell bodies is highly symmetric between the superior and inferior hemi-field of the retina in normal eyes. [9] Glaucomatous changes of the retinal layers are often asymmetric between the upper and lower hemi-fields, as in comparison between the right and left eyes. Several studies have reported that the analysis evaluating the difference in macular total retinal thickness between retinal hemispheres (Posterior Pole Asymmetry Analysis; Heidelberg Engineering, Heidelberg, Germany) is an indicator of glaucomatous change. [8,10–12] In fact, a recent report suggested that the macular retinal layer thickness asymmetry index, which is calculated by measuring segmented layers in the macular inner retina, can be an early indicator of glaucomatous damage. [13] Similarly, our group recently reported the automated detection of hemifield difference in GCIPL thickness map may be effective for glaucoma diagnosis. [14]

Recently, a new-generation OCT, swept-source OCT has been introduced for evaluation of glaucomatous structural changes. Swept-source OCT uses longer wavelength (1050 nm) for imaging which, in comparison to spectral domain OCT, allows greater detailing of the deep ocular structures although with a reduction in the contrast of retinal layers. Previous studies have demonstrated the utility of swept-source OCT for evaluation of deep ocular tissues, such as the choroid and lamina cribrosa. [15–17] Additionally, the faster scan speed of swept-source OCT enables investigators to obtain high-quality wide-angle scan images of large areas of the posterior pole. A recent study reported similar diagnostic accuracies from swept-source OCT and spectral-domain OCT. [18] On this basis, we speculated that combining high-quality wide-angle swept-source OCT scans with conventional asymmetry analysis methods might improve the utility of asymmetry analysis in the evaluation of glaucoma.

The current study was designed to report inter-ocular and inter-hemispheric asymmetry analysis on macular inner retinal layers using swept-source OCT. We analyzed the thicknesses of the RNFL, GCIPL, ganglion cell complex, and total retina with the asymmetry analysis method, and evaluated its effectiveness for distinguishing glaucoma patients from healthy subjects.

## Materials and Methods

### Subjects

This study was an observational, cross-sectional study, based on the Swept-source Optical Coherence Tomography Wide-view Study, an ongoing prospective study of glaucoma patients and healthy volunteers at the Glaucoma Clinic of Seoul National University Hospital. Each subject was informed of the nature of the study, and written informed consent was obtained from each participant after study approval was received from the Institutional Review Board of Seoul National University Hospital. The study protocol complied with the Declaration of Helsinki.

Eyes were randomly selected from a database of open angle glaucoma patients and healthy subjects. Eligibility was determined for each subject by means of a complete ophthalmological examination, including measurement of visual acuity, refraction, slit-lamp examination, intraocular pressure (IOP) measurement by Goldmann applanation tonometry, central corneal thickness measurement, dilated fundus examination, red-free photography, standard automated perimetry (Swedish Interactive Threshold Algorithm [SITA] standard 30–2 test with Humphrey Field Analyzer), and swept-source OCT (DRI OCT-1 Atlantis, Topcon). The inclusion criteria were as follows: age between 20 and 79 years; best-corrected visual acuity of ≥20/40; refractive error within ±6.00 diopters equivalent sphere and ±3.00 diopters astigmatism; less than 2.00 diopters anisometropia; an open anterior chamber angle at the initial exam, and high-quality fundus images.

The main exclusion criteria were contraindication of dilation or intolerance to topical anesthetics or mydriatics; intraocular surgery in the study eye (except uncomplicated cataract surgery performed more than 1 year before enrollment); history or evidence of retinal disease such as diabetic retinopathy, macular edema, or other vitreoretinal disease, and evidence of non-glaucomatous optic nerve abnormality. Eyes with consistently unreliable Humphrey visual-field test results (defined as false negative >15%, false positive >15%, and fixation losses >20%) were also excluded from the study. In cases in which both eyes of a normal healthy subject were eligible for the study, one eye was chosen randomly for inclusion.

Glaucomatous eyes were defined as those with glaucomatous visual-field defect confirmed by 2 reliable visual field examinations and by the presence of glaucomatous optic neuropathy. Glaucomatous visual field defects were defined based on the Hodapp-Parrish-Anderson criteria. [19] Glaucomatous optic neuropathy was defined as neuroretinal rim thinning, notching, excavation, or RNFL defect with corresponding visual field deficit. Color disc photography and red-free RNFL images were evaluated independently by 2 observers in a random order and in masked fashion. The presence of glaucomatous optic neuropathy was determined by consensus between the 2 glaucoma specialists (S-Y.L. and J.W.J.). The IOP level was not included in the definition of glaucomatous eyes. Furthermore, gonioscopic examination was performed to exclude eyes affected with angle closure glaucoma. The normal healthy control eyes had an IOP of ≤21 mmHg with no history of elevated IOP, absence of glaucomatous optic neuropathy, absence of RNFL defect on red-free fundus photography, and nonexistent glaucomatous visual field defect upon the standard automated perimetry.

### Optical coherence tomography scanning procedure

Pupils were dilated with tropicamide 1% and phenylephrine 2.5% drops. All imaging procedures were performed using swept-source deep-range imaging OCT (DRI OCT-1, Topcon, Tokyo, Japan) and spectral domain OCT (Cirrus high-definition OCT, Carl Zeiss Meditec, Dublin, CA, USA). Using swept-source OCT, the wide-angle scan, which entailed three-dimensional 12 × 9 mm scans centered on the posterior pole, were acquired from both eyes of each subject. The 12 × 9 mm scan comprises 256 B-scans, each in turn including 512 A-scans for a total of 131,072 axial scans per volume. The swept-source OCT displayed a lateral resolution of 20 μm, in-depth resolution of 2.6 μm, and a scan speed of 100,000 A-scans per second. The thicknesses of the four retinal layers including RNFL, GCIPL, ganglion cell complex, and total retina were measured automatically using the internal DRI OCT segmentation software algorithms. Only high-quality scans were used for analysis, which were defined as scans with signal strength ≥ 6 and the absence of involuntary saccade or blinking artifacts. The accuracies of the segmentation algorithms were reviewed by one masked reviewer (E.K.L.). In a wide-angle scan, the DRI-OCT software calculates the average thickness for each 0.04 mm^2^ grid square of the 12 × 9 mm scan. The thicknesses of the four retinal layers were obtained, using the manufacturer's OCT-Batch utility, within a 6.2 × 6.2 mm macular area centered on the foveal pit, which was divided into a 31 × 31 grid of 200 × 200 μm squares. The location of the foveal pit was identified manually by reviewing the fundus photograph.

Using Cirrus OCT, two scans including one macular scan centered on the fovea (macular cube 200 × 200 protocol) and one peripapillary RNFL scan centered on the optic disc (optic disc cube 200 × 200 protocol), were acquired. The Cirrus OCT had a transverse resolution of 15 μm and an axial resolution of 5 μm. The scan speed of the Cirrus OCT was 27,000 to 68,000 A-scans per second. The macular GCIPL and peripapillary RNFL thicknesses were measured using the internal RNFL and ganglion cell analysis algorithm. Only high-quality scans were used for analysis, which were defined as scans with signal strength ≥6 and the absence of involuntary saccade or blinking artifacts. The accuracies of the segmentation algorithms were again reviewed by the same masked reviewer (E.K.L.).

### Asymmetry calculation and drawing of asymmetry maps

For the analysis, either eye was chosen randomly between the right and left eyes in healthy control subjects. The target eye referred to the selected eye in each healthy control. In the glaucoma group, the target eye referred to the eye with glaucoma, as defined above. In cases in which both eyes of a glaucoma patient were eligible, one eye was chosen randomly for analysis.

In the inter-ocular asymmetry analysis, the thicknesses of the retinal layers on 31 × 31 grid centered on the foveal pit were compared between the subject eye and the contra-lateral eye by the superpixel-to-superpixel comparison method. For each of the corresponding superpixels between the eyes, the thickness difference was calculated as follows: inter-ocular thickness difference of the superpixel of the target eye = thickness of the target superpixel of the subject eye − thickness of the corresponding superpixel of the contra-lateral eye. Corresponding superpixels was defined as mirrored pairs of superpixels between the right and left eyes. After that, only the negative values, representing relative decreases compared with the corresponding superpixels, were selected for the analysis, and the positive values were excluded. The negative differences were displayed on a gray-scale asymmetry map. Black superpixels were defined as thickness decreases over the relevant cut-off values. The cut-off value for black superpixels in the GCIPL thickness analysis was 20 μm, and for the RNFL, ganglion cell complex, and total retinal analyses, 40 μm. These criteria were selected based on findings reported by previous studies using spectral-domain OCT. Jeoung et al. [20] reported that the mean of average GCIPL thickness was 18.4 to 24.9 μm thinner in early and moderate-to-severe glaucoma patients than in healthy subjects (normal: 80.4 ± 6.7 vs. early glaucoma: 72.0 ± 7.4, moderate-to-severe glaucoma: 65.5 ± 10.0 μm). Yamada et al. [13] reported that difference of average ganglion cell complex thickness between normal and glaucoma patient groups was 30.5 μm (105.7 ± 8.8 vs. 75.2 ± 10.9 μm). Since we calculated values in minutely divided superpixels, the criteria were determined conservatively.

**Fig 1:**
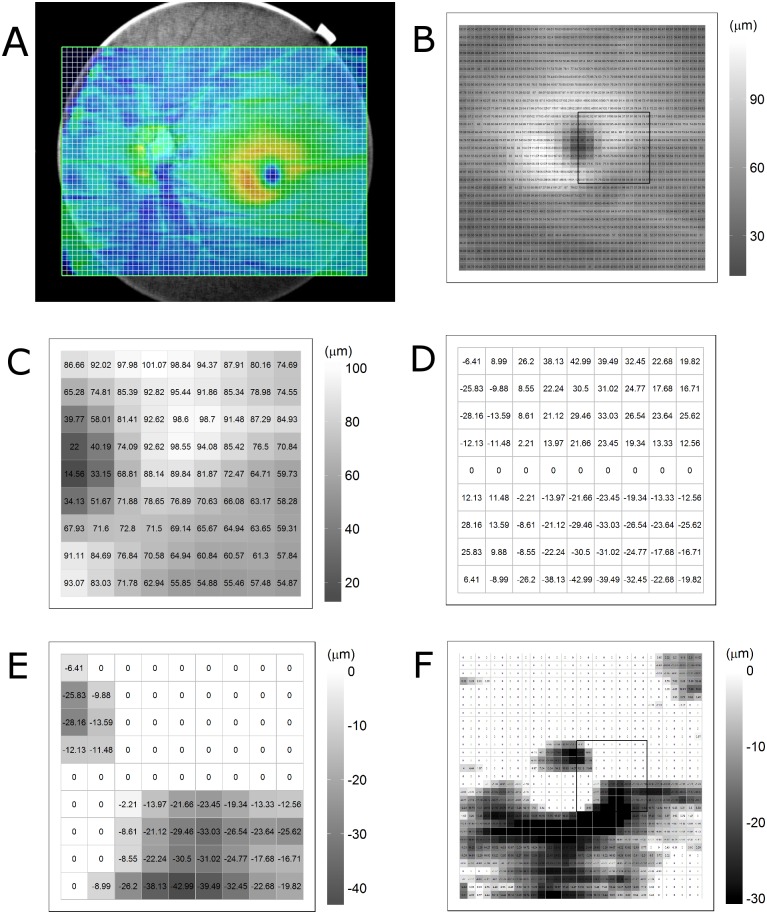
Methods of data processing in the inter-hemisphere asymmetry analysis in present study. (A) Ganglion cell-inner plexiform layer (GCIPL) thickness map drawn from three-dimensional scans of 12 mm width using swept-source optical coherence tomography. (B) GCIPL thickness map of 6.2 × 6.2 mm macular area divided into a 31 × 31 grid of 200 × 200 μm squares. The black square of 1.8 × 1.8 mm area near the foveal pit was marked as an example for detailed description of the calculation process. (C) GCIPL thickness map in the sample square is shown. (D) Map of inter-hemispheric differences. The thickness difference was calculated for each of the corresponding superpixels across the horizontal reference line drawn through foveal pit center. (E) Only negative values, representing relative decreases compared with corresponding superpixels, were selected for analysis, and positive values were excluded. The negative differences were displayed on a gray-scale asymmetry map. (F) Inter-hemispheric GCIPL asymmetry map of 6.2 × 6.2 mm macular area.

In the inter-hemispheric asymmetry analysis, the thicknesses of the retinal layers on the 31 × 31 grid centered on the foveal pit were split into superior and inferior hemi-fields based on a horizontal line drawn through foveal pit center. For each of the corresponding superpixels across the horizontal reference line, the thickness difference was calculated as follows: the inter-hemispheric thickness difference of the superpixel in superior hemi-field = thickness of the target superpixel in superior hemi-field − thickness of the corresponding superpixel in inferior hemi-field. Conversely, the inter-hemispheric thickness difference of the superpixel in inferior hemi-field = thickness of the target superpixel in inferior hemi-field − thickness of the corresponding superpixel in superior hemi-field. Corresponding superpixels was defined as mirrored pairs of superpixels across the horizontal reference line. Subsequently, only the negative values, representing relative decreases compared with the corresponding superpixels, were selected for the analysis, and the positive values were excluded. The negative differences were displayed on a gray-scale asymmetry map. This method of data processing was demonstrated in Fig 1. Black superpixels, as in the inter-ocular analysis, were defined as thickness decreases over the relevant cut-off values. The cut-off value also was the same as in the inter-ocular analysis.

### Statistical analysis

Statistical analyses were performed with SPSS Statistics (version 22.0; IBM, Armonk, NY, USA) and the open-source R program (R Development Core Team, Vienna, Austria). For all of the analyses, a *P* value < .05 was considered statistically significant. The values from the demographic characteristics of the study subjects were compared using Student *t* test and chi-square test. The average values of the negative thickness differences from the asymmetry calculation process were compared between the normal and glaucoma groups by Mann-Whitney test. The numbers of black superpixels on the asymmetry maps were also compared between the normal and glaucoma groups. Receiver operating characteristic (ROC) curves were used to describe the diagnostic utility of inter-ocular and inter-hemispheric thickness differences for differentiation of glaucomatous from normal eyes. An area under the ROC curve (AUROC) of 1.0 represented perfect discrimination, whereas an AUROC of 0.5 represented a chance of discrimination. In the ROC curves analysis for differentiation between bilateral glaucoma group and normal group, the eyes which were selected in the ROC analysis for differentiation between total glaucoma group and normal group were used.

## Results

### Subject characteristics

Data from 70 normal healthy subjects and 62 glaucoma patients were included in this study. The demographic characteristics of the normal and glaucomatous eyes are provided in Table 1. The mean ± standard deviation age was 55.2 ± 12.3 years among the glaucoma patients, and 58.4 ± 12.6 years among the normal healthy subjects, and these results did not represent a significant difference between the groups (*P* = .139). Since the glaucoma patients had been receiving IOP-lowering medication, the mean IOP of glaucomatous eyes was lower than that of normal eyes at examination (*P* = .025). Eyes in the normal control group and glaucoma patient group were mildly myopic; refractive error and axial length showed no statistically significant difference between the two groups. The results of a Humphrey visual-field C30-2 test showed significantly lower mean deviation, higher pattern standard deviation, and lower visual-field index in the glaucomatous eyes than in the healthy eyes (all *P* < .001).

**Table 1:** Demographic Characteristics of Study Subjects.

	Normal Control (n = 70 eyes)	Glaucoma Patient (n = 62 eyes)	P value
Age, year	58.4 ± 12.6	55.2 ± 12.3	0.139*
Female, n (%)	40 (57.1)	41 (66.1)	0.290^†^
Laterality, right, n (%)	41 (58.6)	34 (54.8)	0.666^†^
IOP, mmHg	13.60 ± 2.42	12.69 ± 2.18	0.025*
Central corneal thickness, μm	543.97 ± 36.17	539.04 ± 38.16	0.448*
Spherical equivalent, diopter	-1.32 ± 2.91	-1.90 ± 3.10	0.270*
Axial length, mm	23.85 ± 1.07	24.26 ± 1.41	0.099*
MD, dB	-0.60 ± 1.74	-7.18 ± 7.36	<0.001*
PSD, dB	2.08 ± 0.83	9.05 ± 4.48	<0.001*
VFI, %	99.10 ± 1.23	77.02 ± 21.50	<0.001*
Average RNFL thickness, μm	90.43 ± 9.69	69.76 ± 12.26	<0.001*
Average GCIPL thickness, μm	79.79 ± 7.48	68.47 ± 9.21	<0.001*

SD = standard deviation; IOP = intraocular pressure. MD = mean deviation; PSD = pattern standard deviation; VFI = visual field index; RNFL = retinal nerve fiber layer; GCIPL = ganglion cell-inner plexiform layer.

Data except sex and laterality are presented as mean ± standard deviation.

RNFL and GCIPL thickness values were measured by Cirrus OCT.

MD, PSD, and VFI values were measured by Humphery C30-2 visual field test.

* Student *t* test (two-tailed)

^†^ Chi-square test.

### Average negative differences and number of black superpixels

The average negative differences in the macular inner retinal layer thicknesses between the normal control and glaucoma groups are shown in Table 2. The average negative inter-ocular difference in GCIPL thickness was lower in the glaucomatous eyes than in the healthy eyes (glaucoma group: -4.26 ± 2.23 μm, normal group: -2.78 ± 0.97 μm, *P* < .001). The average negative inter-ocular differences in the RNFL, ganglion cell complex, and total retinal thicknesses were also lower in the glaucoma patient group than in the normal healthy group (all P < .001). In the inter-hemispheric analysis, the average negative differences of GCIPL thickness were lower in the glaucomatous eyes than in the healthy eyes (glaucoma group: -4.88 ± 1.46 μm, normal group: -3.43 ± 0.71 μm, *P* < .001). The average negative inter-hemispheric differences in the RNFL, ganglion cell complex, and total retinal thicknesses showed larger values in the glaucomatous eyes than in the healthy eyes (all *P* < .001).

**Table 2:** Average Negative Differences of Thicknesses in Macular Inner Retinal Layers and Numbers of Black Superpixels in Asymmetry Map in Normal Subjects and Glaucoma Patients.

Macular inner retinal layer		Average negative difference of thickness*		Number of black superpixels^†^	
		Inter-ocular difference	Inter-hemisphere difference	Inter-ocular asymmetry map	Inter-hemisphere asymmetry map
RNFL	Normal, μm	-2.21 ± 1.66	-3.58 ± 1.29	0.63 ± 2.18	6.70 ± 12.39
	Glaucoma, μm	-7.85 ± 5.17	-8.16 ± 3.92	27.97 ± 41.91	39.21 ± 42.23
	P value^‡^	<0.001	<0.001	<0.001	<0.001
GCIPL	Normal, μm	-2.78 ± 0.97	-3.43 ± 0.71	12.04 ± 14.47	18.31 ± 18.13
	Glaucoma, μm	-4.26 ± 2.23	-4.88 ± 1.46	38.26 ± 48.27	55.87 ± 43.18
	P value^‡^	<0.001	<0.001	<0.001	<0.001
GCC	Normal, μm	-2.75 ± 1.78	-4.12 ± 0.95	1.10 ± 3.01	4.89 ± 9.47
	Glaucoma, μm	-9.25 ± 6.83	-9.90 ± 4.80	48.21 ± 74.35	68.45 ± 74.54
	P value^‡^	<0.001	<0.001	<0.001	<0.001
Total retina	Normal, μm	-3.38 ± 2.16	-5.86 ± 1.70	1.93 ± 4.47	9.11 ± 20.81
	Glaucoma, μm	-8.68 ± 6.08	-11.28 ± 5.29	36.71 ± 63.41	88.35 ± 86.99
	P value^‡^	<0.001	<0.001	<0.001	<0.001

RNFL = retinal nerve fiber layer; GCIPL = ganglion cell-inner plexiform layer; GCC = ganglion cell complex.

Data are presented as mean ± standard deviation.

* Positive differences compared with corresponding superpixels were excluded, because the negative values represent relative decrease of thickness.

^†^ Black superpixels were defined as decrease of thickness over cut-off value.

^‡^ Mann-Whitney test.

**Fig 2:**
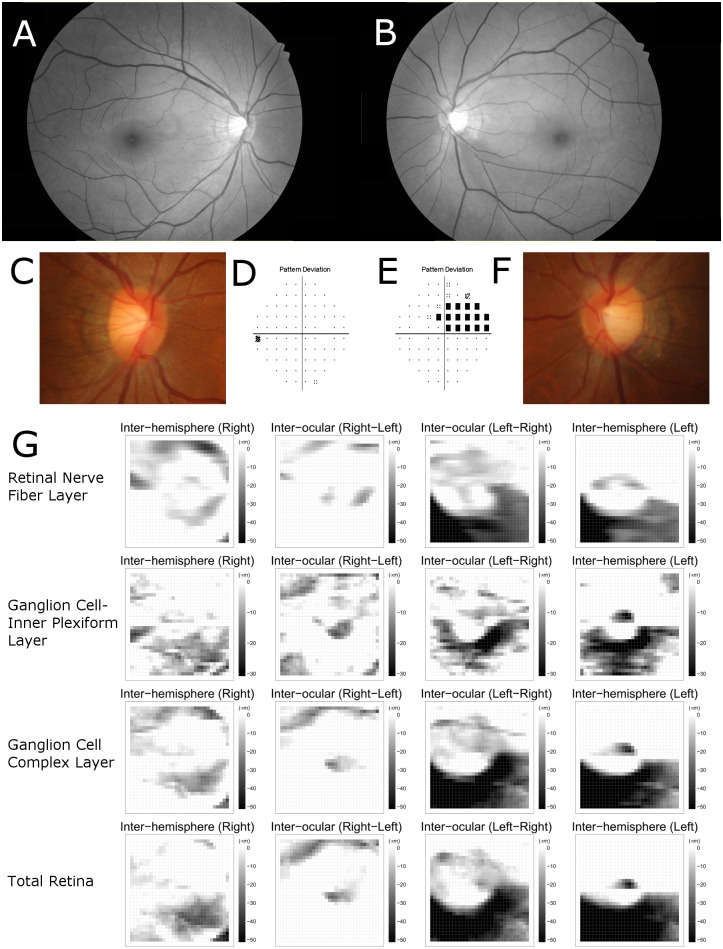
A representative case of glaucoma patient. A 45-year-old woman was evaluated for possible open angle glaucoma. Best corrected visual acuities were 20/20 in both eyes and intraocular pressures were 15 mmHg in both eyes. (A,B) Retinal nerve fiber layer (RNFL) photography indicates inferotemporal RNFL thinning in the left eye. (C,D,E,F) Optic disc of the left eye shows signs of inferotemporal disc notching. The results of Humphrey visual field testing were normal in the right eye and superior defect in the left eye. The mean deviation was 0.69 dB in the right eye and -3.80 dB in the left eye. The visual field index was 100% and 84% for the right and left eye, respectively. (G) Asymmetry maps from inter-ocular and inter-hemispheric asymmetry analyses showing a cluster of black superpixels corresponding with inferotemporal RNFL defect in left eye. The inter-hemispheric and inter-ocular asymmetry thickness maps of RNFL, ganglion cell-inner plexiform layer (GCIPL), ganglion cell complex and total retina were arrayed in a 4 × 4 matrix form. The maps of the first and fourth columns were constructed from inter-hemispheric asymmetry analyses of the right and left eye, respectively. The maps of the second and third columns were obtained from inter-ocular asymmetry analyses of the right and left eye, respectively. The rows of the maps are arrayed in the order RNFL, GCIPL, ganglion cell complex, total retina.

**Fig 3:**
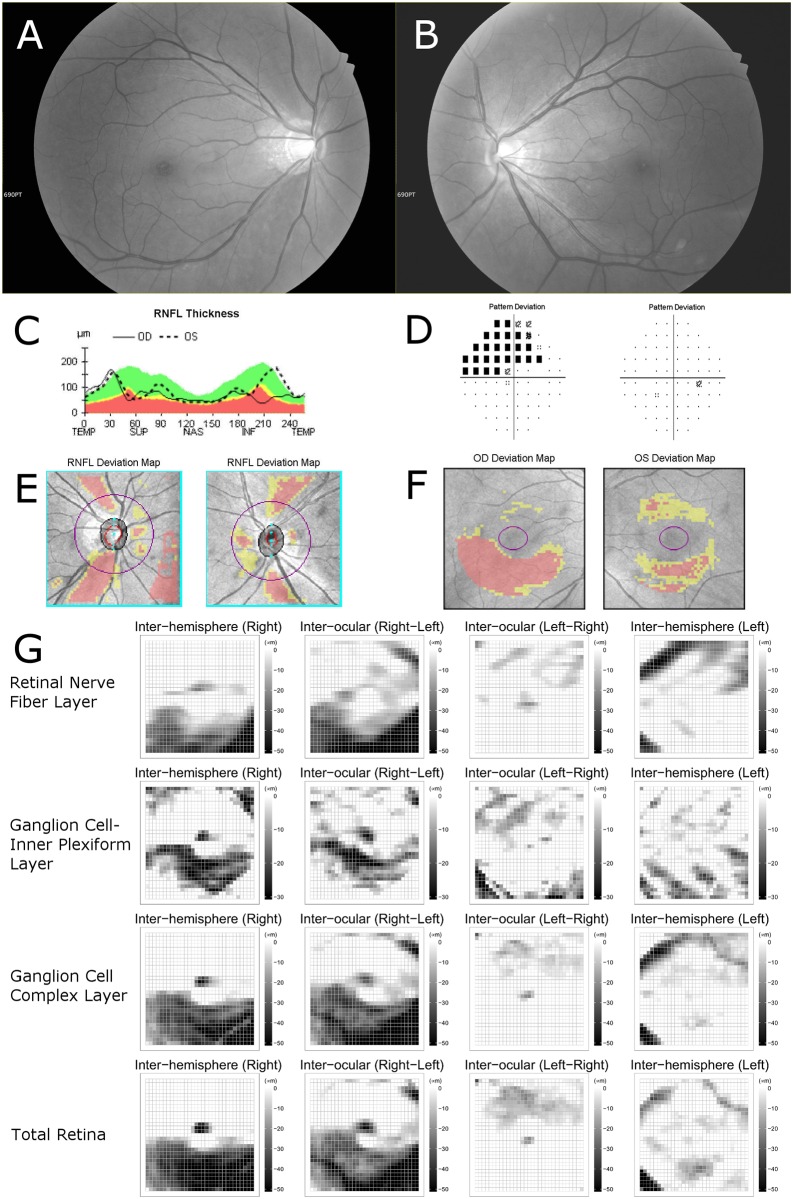
A representative case of unilateral glaucoma patient with false positive findings on conventional deviation map. A 58-year-old man was evaluated for open angle glaucoma. Best corrected visual acuities were 20/20 in both eyes and intraocular pressures were 18 mmHg in the right eye and 16 mmHg in the left eye. The refractive errors were -5.50 diopters in both eyes. (A,B) Retinal nerve fiber layer (RNFL) photography presents inferotemporal RNFL thinning in right eye. (C) Topographic profile of peripapillary RNFL thickness measured by Cirrus OCT presented temporal deviation of peaks of RNFL thicknesses. (D) The results of Humphrey visual-field testing were superior defect in right eye and normal in left eye. (E) The peripapillary RNFL thickness deviation map of Cirrus optical coherence tomography (OCT) showed abnormal color-coded area consistent with superotemporal and inferotemporal RNFL thinning in both eyes. (F) The ganglion cell analysis deviation map of Cirrus OCT also showed abnormal color-coded area in the inferior macular area of the right eye and in the superior and inferior macular area of the left eye. The abnormal color-coded areas of the left eye were considered as false-positive because of normal findings on RNFL photography and visual-field testing. (G) Inter-hemispheric and inter-ocular asymmetry thickness maps of RNFL, ganglion cell-inner plexiform layer, ganglion cell complex and total retina were arrayed in 4 × 4 matrix form in same order as Fig 2G. The asymmetry maps from the inter-ocular and inter-hemispheric asymmetry analyses showed a cluster of black superpixels corresponding to an inferotemporal RNFL defect in the right eye. However, in the left eye, the number of black superpixels was small and the map showed only a scattered distribution of black superpixels. The asymmetry maps suggest that this case has significant structural changes only in the right eye, which corresponded well with the results for RNFL photography and visual-field testing.

Table 2 also shows the numbers of black superpixels on the asymmetry map. The average number of black superpixels on the inter-ocular asymmetry map of GCIPL thickness was larger in the glaucoma group than in the normal control group (glaucoma group: 38.26 ± 48.27, normal group: 12.04 ± 14.47, *P* < .001). The average numbers of black superpixels on the inter-ocular asymmetry map of RNFL, ganglion cell complex, and total retina thicknesses were also larger in the glaucoma group than in the normal group (all *P* < .001). Similarly, on the inter-hemispheric asymmetry map of RNFL, GCIPL, ganglion cell complex, and total retinal thicknesses, all of the average numbers of black superpixels were larger in the glaucoma group than in the normal group (all *P* < .001). The inter-ocular and inter-hemispheric asymmetry maps of representative cases including glaucoma patients are shown in Figs 2 and 3.

### Area under receiver operating characteristic curve

Table 3 shows the AUROCs of the average negative thickness differences among the four macular inner layers. The AUROCs ranged from 0.748 to 0.894; the inter-hemispheric ganglion cell complex thickness (AUROC = 0.894), inter-ocular RNFL thickness (AUROC = 0.868), and inter-hemispheric RNFL thickness (AUROC = 0.862) were best for discriminating between glaucomatous and healthy eyes. The AUROCs from the ROC analysis were also performed on the bilateral glaucoma patients and normal controls. The AUROCs ranged from 0.720 to 0.886; the inter-hemispheric ganglion cell complex thickness (AUROC = 0.886), inter-hemispheric RNFL thickness (AUROC = 0.857), and inter-ocular RNFL thickness (AUROC = 0.847) were best for discriminating between glaucomatous eyes and healthy eyes in cases of bilateral glaucoma.

**Table 3:** Area Under Receiver Operating Characteristic Curve Values of Average Negative Differences of Thicknesses in Macular Inner Retinal Layers for Diagnosis of Glaucoma.

Macular inner retinal layer	AUROC of average negative difference of thickness			
	Total glaucoma (62 eyes) vs normal (70 eyes)		Bilateral glaucoma (52 eyes) vs normal (70 eyes)	
	Inter-ocular asymmetry map	Inter-hemisphere asymmetry map	Inter-ocular asymmetry map	Inter-hemisphere asymmetry map
RNFL (95% CI)	0.868 (0.802, 0.934)	0.862 (0.796, 0.928)	0.847 (0.771, 0.923)	0.857 (0.784, 0.930)
GCIPL (95% CI)	0.748 (0.665, 0.831)	0.816 (0.738, 0.895)	0.720 (0.628, 0.811)	0.805 (0.718, 0.893)
GCC (95% CI)	0.850 (0.778, 0.923)	0.894 (0.830, 0.958)	0.825 (0.742, 0.909)	0.886 (0.813, 0.959)
Total retina (95% CI)	0.809 (0.730, 0.888)	0.830 (0.757, 0.903)	0.798 (0.712, 0.884)	0.821 (0.741, 0.901)

AUROC = area under receiver operating characteristic curve; RNFL = retinal nerve fiber layer; CI = confidence interval; GCIPL = ganglion cell-inner plexiform layer; GCC = ganglion cell complex.

The AUROCs of the black superpixel numbers on the asymmetry maps of four macular inner layers are listed in Table 4. The AUROCs ranged from 0.709 to 0.840; the inter-hemispheric ganglion cell complex thickness (AUROC = 0.840), inter-ocular total retinal thickness (AUROC = 0.839), and inter-hemispheric total retinal thickness (AUROC = 0.831) were best for discriminating between glaucomatous and healthy eyes. The AUROCs from the ROC analysis performed on bilateral glaucoma patients and normal controls were also presented in Table 4. The AUROCs ranged from 0.676 to 0.822.

**Table 4:** Area Under Receiver Operating Characteristic Curve Values of Numbers of Black Superpixels in Asymmetry Map for Diagnosis of Glaucoma.

Macular inner retinal layer	AUROC of number of black superpixels			
	Total glaucoma (62 eyes) vs normal (70 eyes)		Bilateral glaucoma (52 eyes) vs normal (70 eyes)	
	Inter-ocular asymmetry map	Inter-hemisphere asymmetry map	Inter-ocular asymmetry map	Inter-hemisphere asymmetry map
RNFL (95% CI)	0.808 (0.741, 0.875)	0.761 (0.681, 0.841)	0.772 (0.696, 0.848)	0.745 (0.656, 0.833)
GCIPL (95% CI)	0.709 (0.620, 0.798)	0.779 (0.693, 0.865)	0.676 (0.578, 0.773)	0.760 (0.664, 0.856)
GCC (95% CI)	0.833 (0.763, 0.902)	0.840 (0.771, 0.908)	0.801 (0.721, 0.881)	0.821 (0.743, 0.898)
Total retina (95% CI)	0.839 (0.774, 0.905)	0.831 (0.759, 0.904)	0.822 (0.749, 0.895)	0.813 (0.731, 0.896)

AUROC = area under receiver operating characteristic curve; RNFL = retinal nerve fiber layer; CI = confidence interval; GCIPL = ganglion cell-inner plexiform layer; GCC = ganglion cell complex.

**Fig 4:**
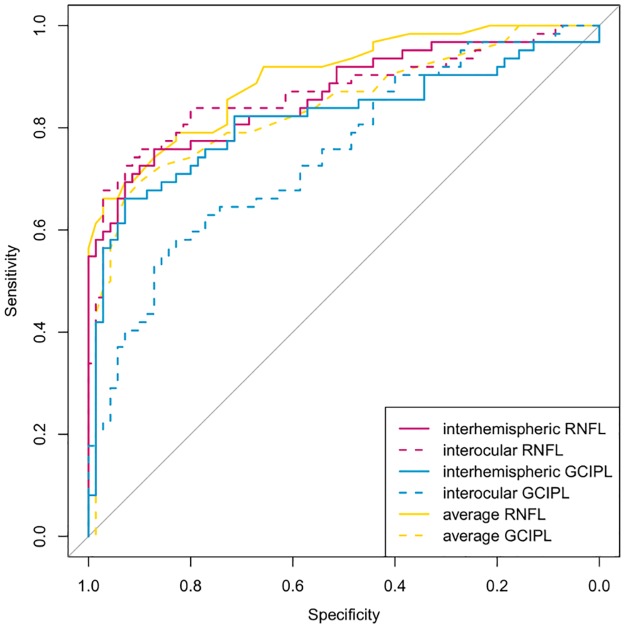
Receiver operating characteristic curves of six parameters for glaucoma diagnosis. The parameters are the inter-hemispheric negative difference of retinal nerve fiber layer (RNFL) and ganglion cell-inner plexiform layer (GCIPL) thicknesses, inter-ocular negative difference of RNFL and GCIPL thicknesses, and average RNFL and GCIPL thicknesses. The average RNFL and GCIPL thicknesses were measured using Cirrus optical coherence tomography.

The AUROC of the average RNFL thickness measured by Cirrus OCT was 0.899 (95% confidence interval: 0.847 to 0.950) and that of the average GCIPL thickness was 0.839 (95% confidence interval: 0.769 to 0.910). Fig 4 plots the ROC curves of the inter-hemispheric negative differences of RNFL and GCIPL thicknesses, inter-ocular negative difference of RNFL and GCIPL thicknesses, and average RNFL and GCIPL thicknesses. The AUROCs showed no significant difference between the average RNFL thickness and average negative difference of RNFL thicknesses from the inter-hemispheric and inter-ocular asymmetry analysis (*P* = .414 and *P* = .400, respectively).

## Discussion

The main objective of the current study was to develop an asymmetry analysis of the thicknesses of macular inner retinal layers including the RNFL, GCIPL, ganglion cell complex, and total retina measured by swept-source OCT. The inter-ocular and inter-hemispheric negative differences from asymmetric analysis showed statistically significant difference between glaucoma patients group and normal control group. Then, based on the ROC analysis, we evaluated the performance of this asymmetry analysis method for diagnosis of glaucoma.

Our results were found to be consistent with those of previous studies on the various asymmetry analysis methods. Um et al. [11] showed that the sensitivity of the modified macular hemi-field test using data from posterior pole asymmetry analysis (PPAA) was significantly greater than that of RNFL measurement in their early glaucoma group. Seo et al. [12] reported that PPAA detects localized RNFL defects with high sensitivity and specificity based on the analysis of the number of black cells in PPAA asymmetry maps, and the AUROC of PPAA was 0.958 ± 0.013. Sullivan-Mee et al. [8] reported an AUROC of inferior/superior macular thickness difference of 0.860. Recently, Yamada et al. [13] developed the method of calculating a value called the asymmetry index and reported AUROCs of novel index in macular retinal layers including RNFL, ganglion cell layer, and ganglion cell complex ranged from 0.861 to 0.998. We recently reported that AUROCs of the automated detection of hemifield difference on GCIPL thickness map from Cirrus OCT ranged from 0.962 to 0.967. [14] With regard to the use of the inter-ocular asymmetry, previously Sullivan-Mee et al. [8] also reported an AUROC for inter-eye difference in the total macular thickness of 0.913. An earlier study [7] conducted by our group reported AUROCs of inter-ocular difference of macular GCIPL thickness ranging from 0.730 to 0.820 based on Cirrus high-definition OCT. Although direct comparison is not feasible due to the different subject populations, the results of the present study are comparable with those of the relevant previous studies on inter-hemispheric asymmetry and inter-ocular asymmetry.

The asymmetry analyses in several previous studies were based on following assumptions. First, inter-ocular and inter-hemispheric anatomic symmetry are preserved in normal healthy eyes. Second, although glaucoma is generally a bilateral and bi-hemispheric disease, it often shows asymmetric features at diagnosis and during progression. Therefore, significant asymmetry when compared with normal eyes may indicate glaucomatous damage. We extended this idea and performed a topographical analysis of the segmented macular inner layers within minutely divided superpixels in the expanded macular area.

In this study, only negative values were selected as relative changes among the values of differences after calculation with subtraction. Considering the progressive nature of glaucoma, the decreases in retinal layer thickness were more significant in the diagnosis of disease. This algorithm was similar to the algorithm used previously in a PPAA of macular retinal thickness. [10–12] Using the fastest scan speed of swept-source OCT, we were able to acquire wide scan images from a single examination. Hence, we believe that combining swept-source OCT scans with conventional asymmetry analysis procedures could be helpful in enhancing the diagnostic capability of these methods. The methodological improvement represented by this study compared with the previous methods can be summarized as follows: (1) we used 931 superpixels (31 × 31 grid) instead of 64 cells (8 × 8 grid) in a previous PPAA, in order to construct a more detailed map; (2) macular layers including macular RNFL, macular GCIPL, and macular ganglion cell complex were additionally analyzed. And with respect to Figs 2 and 3, we expect that the construction of a detailed asymmetry map using data from swept-source OCT will facilitate the topographic analysis of inter-ocular and inter-hemispheric asymmetry. Further studies are needed to extend these approaches.

Among the analytical methods employed to diagnose and evaluate glaucomatous eyes with OCT, the attempt to use the asymmetry analysis might have an advantage over conventional methods. According to previous studies, [21,22] the color-coded thickness map and the color-coded deviation map of RNFL and GCIPL show high diagnostic sensitivity, but at the same time, high false-positive results. The abnormal diagnostic classification of color-coded maps has frequently arisen for healthy subjects showing anatomic variation such as high refractive error, optic disc tilt or torsion, large optic disc area, and large peripapillary atrophy, [23–27] because color-coded maps are drawn by comparing measured data with an internal normative database. We speculated that asymmetry analysis can be helpful for diagnosis and evaluation of these patients, because the analysis proceeds by calculating relative change, specifically by comparing the thicknesses of the target area with those of the corresponding area. On the other hand, asymmetry analysis for glaucoma might have an inevitable disadvantage in the glaucoma patients presenting symmetric damage, such as advanced glaucoma. It should also be noted that asymmetry analysis cannot be applied to subjects with a high possibility of anatomical asymmetry such as anisometropia.

The AUROCs of our asymmetry analysis did not show superior diagnostic performance when compared to RNFL and GCIPL thickness analyses performed using Cirrus OCT. This could be attributed to the limitations of asymmetry analysis algorithm described above. The stage of glaucoma could also be an important factor evaluating the utility of our asymmetry analysis for glaucoma diagnosis. Yamada et al. previously reported the macular retinal layer thickness asymmetry index were higher diagnostic performance in preperimetric and early glaucoma patients. [13] Among 62 glaucoma patients in this study, twenty-nine patients showed early defects, 17 patients showed moderate defects, and 16 patients represents severe defects after the classification based on the Hodapp-Parrish-Anderson criteria. Moreover, the preperimetric glaucoma patients were not included in this study. Further studies will be needed to identify the target population in which the present asymmetry analysis using swept-source OCT will be helpful for the diagnosis of glaucoma.

The present study has several limitations. First, selection bias might have occurred, particularly with regard to the stage of glaucoma. Although we included subjects randomly selected from a database, the relatively small numbers of normal subjects and glaucoma patients made it difficult to perform subgroup analysis for the stage of glaucoma, and different results could be found in different study population. Further studies with larger sample sizes and with various stages of glaucoma are needed to validate our results. Second, our data derived only from Korean subjects, and thus lack ethnic variety. Third, the horizontal line passing through the fovea was used in the inter-hemispheric asymmetry analysis. Use of the fovea-disc line as the axis of symmetry, as in the previous PPAA, might have yielded different results. However, recent report [28] has shown that the horizontal raphe in the temporal macular area was different from the fovea-disc line. Additional studies on the axis of symmetry between the hemi-fields are needed.

Despite its limitations, the present asymmetry analysis of macular inner retinal layers using swept-source OCT showed significant differences between the normal and glaucoma groups. The diagnostic performance of both inter-ocular and inter-hemispheric asymmetry analysis in differentiating glaucoma patients from healthy subjects was found to be comparable to those of previous analysis methods with OCT. These findings suggest that the asymmetry analysis method have promise as an ancillary diagnostic tool for evaluation of glaucomatous damage.

## Supporting Information

S1 File Anonymized raw dataset of the aymmetry analysis. This supplementary material provides the anonymized raw dataset of the asymmetry anaylsis.
(XLSX)
